# Illustrated Neuropathologic Diagnosis of Alzheimer’s Disease

**DOI:** 10.3390/neurolint15030054

**Published:** 2023-07-12

**Authors:** Nicholas Doher, Vahid Davoudi, Shino Magaki, Ryan A. Townley, Mohammad Haeri, Harry V. Vinters

**Affiliations:** 1 Department of Neurology, University of Florida, Gainesville, FL 32611, USA; 2 Department of Pathology and Laboratory Medicine, University of Kansas Medical Center, Kansas City, KS 66160, USA; 3 Department of Pathology and Laboratory Medicine, David Geffen UCLA School of Medicine, Los Angeles, CA 90095, USA; 4 Department of Neurology, University of Kansas Medical Center, Kansas City, KS 66160, USA; 5 The University of Kansas Alzheimer’s Disease Research Center, University of Kansas Medical Center, Fairway City, KS 66205, USA; 6 Department of Neurology, David Geffen UCLA School of Medicine, Los Angeles, CA 90095, USA; 7 Brain Research Institute, David Geffen UCLA School of Medicine, Los Angeles, CA 90095, USA

**Keywords:** Alzheimer’s disease, neurodegeneration, tauopathy, dementia, AD neuropathologic diagnosis

## Abstract

As of 2022, the prevalence of Alzheimer’s disease (AD) among individuals aged 65 and older is estimated to be 6.2 million in the United States. This figure is predicted to grow to 13.8 million by 2060. An accurate assessment of neuropathologic changes represents a critical step in understanding the underlying mechanisms in AD. The current method for assessing postmortem Alzheimer’s disease neuropathologic change follows version 11 of the National Alzheimer’s Coordinating Center (NACC) coding guidebook. Ambiguity regarding steps in the ABC scoring method can lead to increased time or inaccuracy in staging AD. We present a concise overview of how this postmortem diagnosis is made and relate it to the evolving understanding of antemortem AD biomarkers.

## 1. AD Antemortem Biomarkers

The advent of biomarkers has brought about a new era of improving the antemortem diagnostic accuracy of AD [1]. Biomarkers can be subdivided into biofluid-based laboratory tests such as amyloid-β 42 (Aβ_42_) to amyloid-β 40 (Aβ_40_) ratio (Aβ_42/40_), total tau (T-tau), phosphorylated tau (P-tau), and neurofilament light (NfL) and diagnostic imaging, including positron emission tomography (PET) scans targeting glucose metabolism, amyloid-β, or tau deposition in the brain with unique ligands. Together with structural MRI, these biomarkers form the framework of the A/T/N system for antemortem AD diagnosis defined by the National Institute on Aging and Alzheimer’s Association (NIA-AA), where “A” refers to amyloid, “T” refers to tau, and “N” refers to neurodegeneration [2].

The cerebrospinal fluid (CSF) concentration of amyloid-β decreases in patients with AD and correlates with the formation of neuritic plaques in the brain, making this test an effective biomarker for AD pathology [3,4]. Subsequent studies demonstrated that the ratio of Aβ_42_ to Aβ_40_ increases the specificity of predicting AD pathology antemortem [5]. Aβ_42_ to Aβ_40_ ratio (Aβ_42/40_) is decreased in AD patients due to intraparenchymal accumulation [5]. PET scans targeting amyloids correlate with the typical progression of amyloid deposition in different brain regions found at autopsy [6,7,8,9]. In one study, amyloid PET data was used to assess 667 patients, and the study found a regional hierarchy of amyloid deposition that resembled previously defined neuropathologic findings [10]. It is important to note that roughly 30% of cognitively normal patients are amyloid-PET-positive, but it is unclear if this cohort of patients represents preclinical AD or subjects at increased risk for AD [11].

CSF P-tau is relatively specific for AD pathology compared to other neurodegenerative diseases [12] and correlates well with NFT burden [4,13,14,15]. In general, the density of NFTs found at autopsy correlates better with cognitive status than the burden of amyloid-β plaques [16]. In research-oriented clinical practice, the increased levels of CSF T-tau are sensitive for neurodegenerative tauopathies [17], and it has been shown that phosphorylated tau markers such as P-tau181 and P-tau217 are particularly specific for AD pathologic process [18]. With regards to tau PET imaging, the topographic pattern has been shown to correlate with clinical AD progression [19,20,21] and more recently with postmortem Braak staging [22].

NfL is a non-specific marker of neuro-axonal damage and is elevated in the CSF of AD patients [23] as well as patients with dementia with Lewy bodies, Parkinson’s disease dementia, frontotemporal dementia, vascular dementia, Creutzfeldt–Jakob disease, and amyotrophic lateral sclerosis [24,25,26]. High CSF levels of NfL have been shown to predict cognitive decline [27,28] and show a strong correlation with neuro-axonal damage and possibly cognition when measured in the plasma [29,30]. In a small study involving 26 patients, p-tau181 and NfL antemortem biomarkers correlated with postmortem-pathology-proven AD dementia [31]. Successful treatment of neurologic diseases such as multiple sclerosis [32] and spinal muscular atrophy [33] has resulted in reduction in NfL back to normal levels. This provides support for using NfL as a potential future marker of treatment response in neuro-axonal damage. Scans targeting F-18 fluorodeoxyglucose (FDG) to examine regional glucose metabolism can be combined with clinical assessment to improve diagnostic accuracy. Bloudek et al. compiled a meta-analysis including 119 studies and found FDG PET alone had a sensitivity of 92% (95% CI 84% to 96%) and specificity of 78% (95% CI 69% to 85%) in discriminating AD from non-AD demented controls [34]. Tripathi et al. found 93.4% concordance by combining clinical diagnosis with FDG PET results [35].

## 2. Diagnosis of Alzheimer’s Disease Neuropathologic Change (ADNC)

The hallmark neuropathologic changes seen in AD are neurofibrillary tangles (NFTs) composed of hyperphosphorylated tau protein aggregates and amyloid-β deposition, most specifically neuritic plaques (NP). Neuritic plaques have been described as a subset of senile plaques in which a central core of amyloid-β deposits is surrounded by a cluster of dystrophic neurites, frequently immunoreactive with phospho-tau antibodies [36,37].

Additional neuropathologic changes include cerebral amyloid angiopathy; neuronal and synaptic loss; granulovacuolar degeneration, which is usually confined to the hippocampus; and neuroinflammation [16]. The postmortem diagnosis of ADNC involves assessing amyloid and tau protein deposition that typically progresses in a predictable manner but differ in their chronology and topography. Tau deposition and progression show the strongest correlation with clinical disease stage [16]. In 2012, postmortem examinations were refined [36] to incorporate semi-quantitative measures of the pathologic hallmarks of AD. This framework utilizes a scoring system to inform the probability that ADNC explains a clinical diagnosis. An ABC scoring system uses four-point scales (0–3) where “A” correlates with “amyloid” or Thal phase, “B” correlates with tau or “Braak” stage, and “C” corresponds to neuritic plaque density in the neocortex based on the Consortium to Establish a Registry for Alzheimer’s Disease (CERAD) score [38,39].

**Figure 1:**
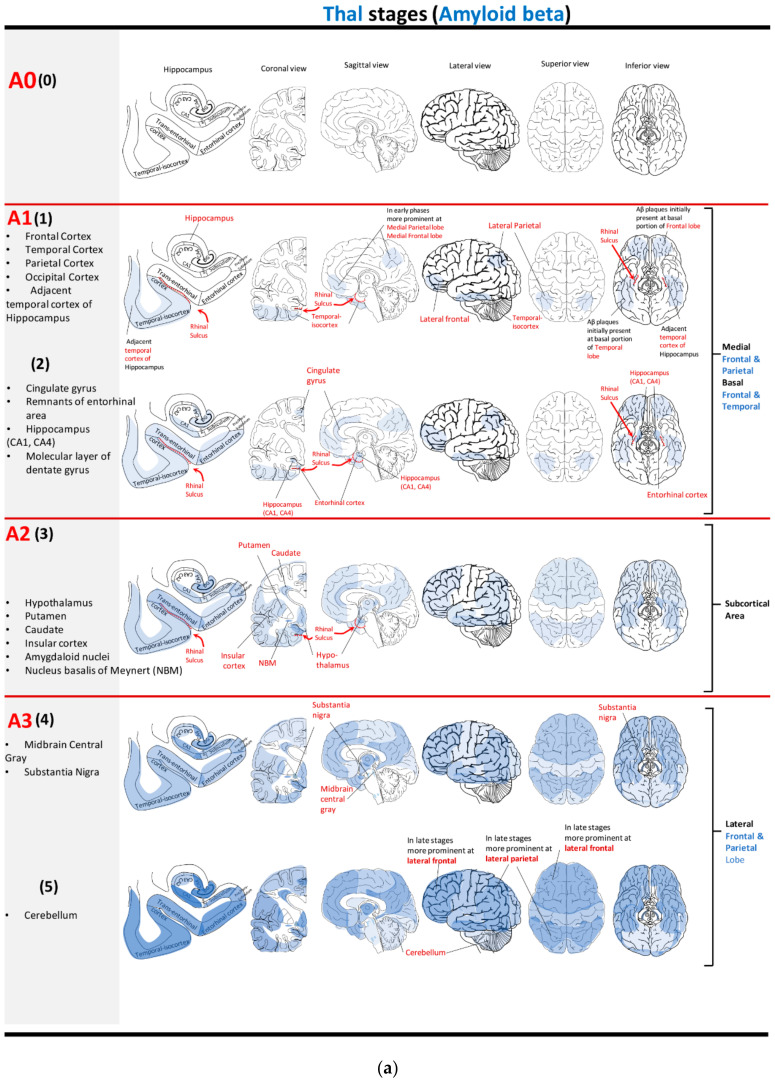

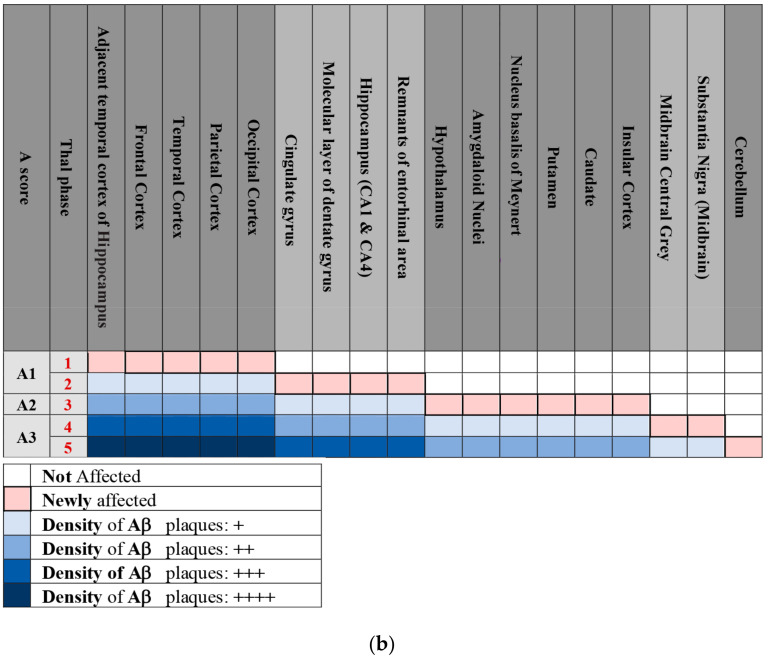
(**a**) Topographical map represents areas of Aβ accumulation (in blue) according to Thal phases and “A” score [38,40,41]. (**b**) Tabulated representation of the data. A1 (1). Aβ plaques begin in the frontal lobe and move posteriorly to affect parietal, temporal, and occipital lobes. They initially accumulate in the basal portion of the frontal and temporal lobes and temporal cortex adjacent to the hippocampus. In the early phases, Aβ plaques can be prominently observed in the medial frontal and medial parietal lobes. A1 (2). In the second phase, they involve cingulate gyrus. Medial temporal regions including entorhinal cortex and hippocampus (CA4 and CA1) are affected as well. A2 (3). Subcortical areas including hypothalamus, putamen, caudate, amygdaloid nuclei, and nucleus basalis of Meynert are affected in this stage. Aβ plaques expand further into the frontal, parietal, temporal, and occipital lobes and affect the insular cortex. A3 (4). Aβ plaques can be observed in the midbrain central gray, locus coeruleus, and substantia nigra. In this phase, the primary motor and sensory cortices are affected. A3 (5). The last phase is characterized by Aβ immunoreactivity in the cerebellum.

The A-score is derived from 5 Thal phases (Figure 1) [38] and staged from 0 to 3 depending on the distribution of APs [38]. It does not discriminate between amyloid plaque morphology or density. An A-score of “0” denotes an absence of amyloid in immunohistochemistry. Amyloid-β deposition begins in the neocortex (Thal phase 1), most commonly in the frontal lobe, and progresses posteriorly, involving association cortices in the temporal, parietal, and occipital lobes. Next, it progresses to the allocortex, including the entorhinal cortex, hippocampal region, and cingulate gyrus (Thal phase 2). Thal phases 1 and 2 are combined into stage A1. When amyloid-β is present in the striatum and the subcortical nuclei, such as the nucleus basalis of Meynert, it is stage “A2” (Thal phase 3). Finally, if amyloid-β is present in the midbrain, pons (Thal phase 4), or the cerebellum (Thal phase 5), the score is “A3”.

**Figure 2:**
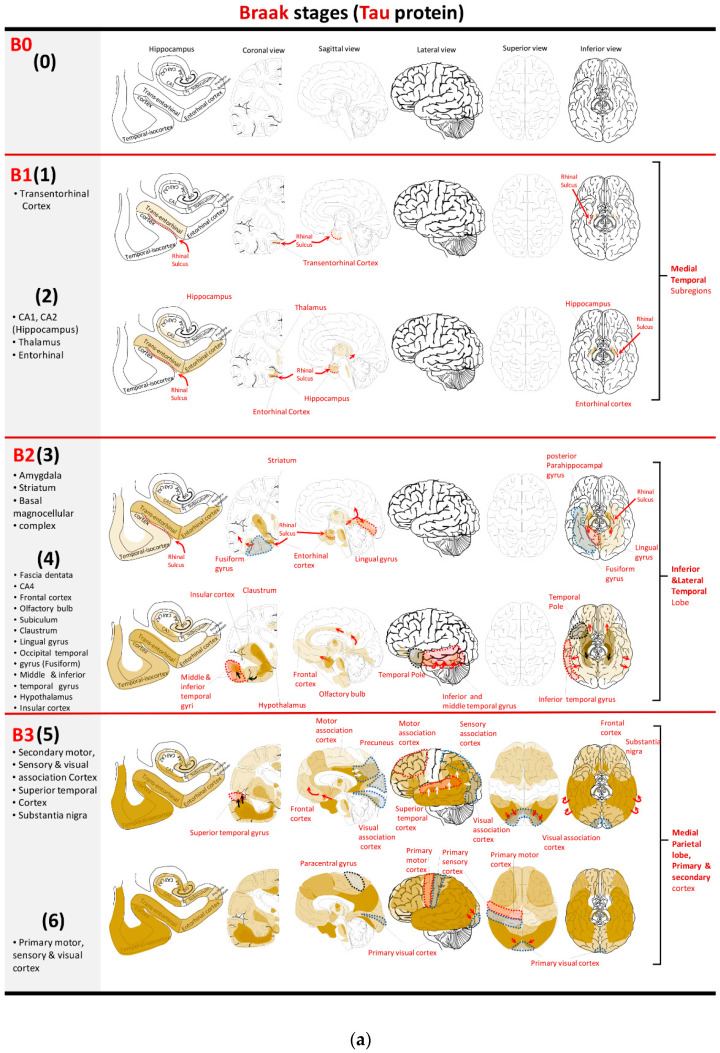

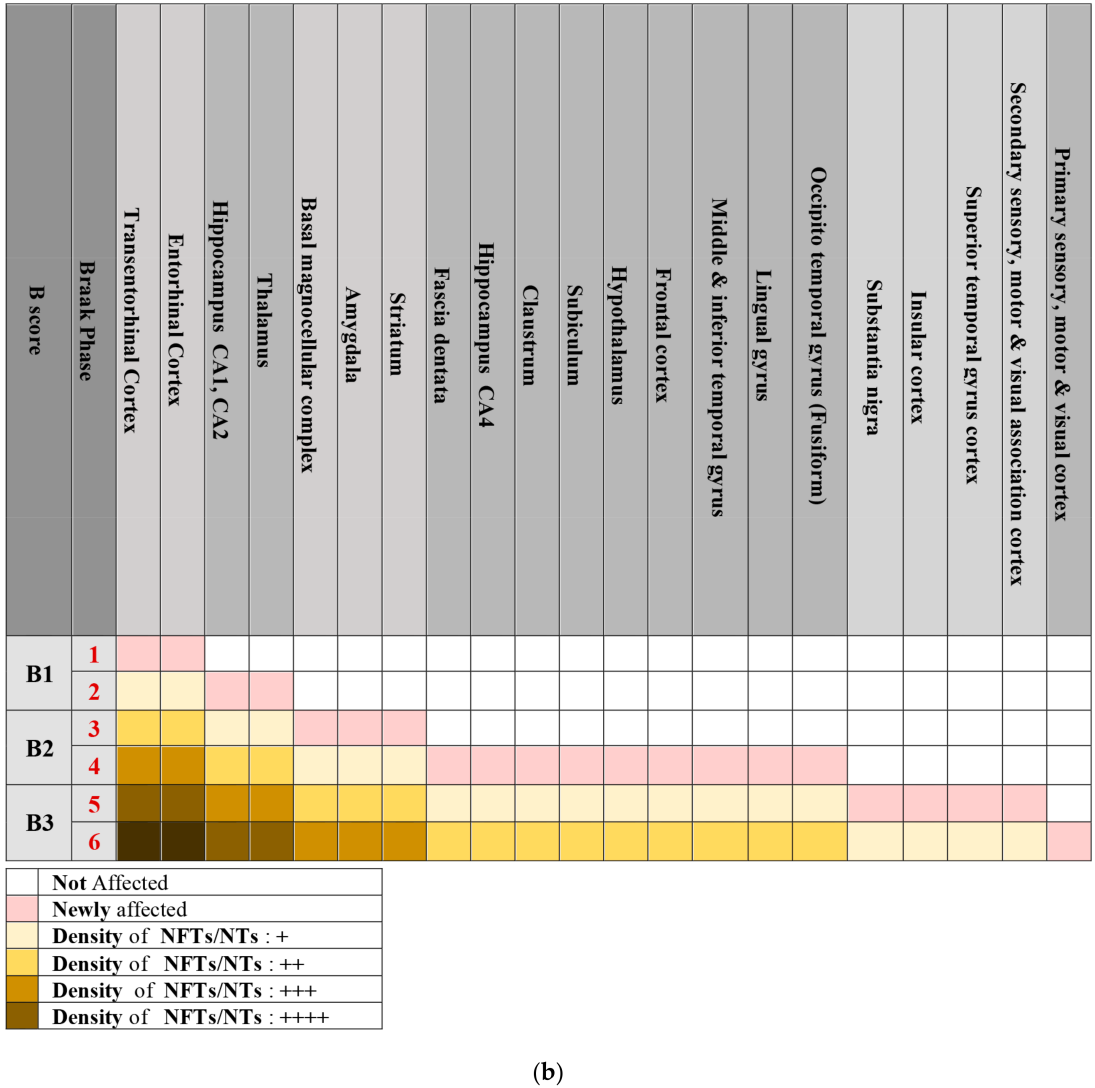
(**a**) Topographical map represents areas of tau aggregation (in gold/brown). (**b**) Tabulated representation of the data. B1 (1). Initial phase of tau aggregation is largely confined to the transentorhinal cortex in the medial temporal area, although there may be minimal involvement of the entorhinal cortex. B1 (2). Lesions extend into the entorhinal cortex and CA1/CA2 of the hippocampus. B2 (3). Subsequently, tau accumulates in the basal magnocellular complex, amygdala, and striatum [42] (while not a constant finding) and spreads posteriorly through the posterior parahippocampal gyrus into lingual gyri and laterally from the medial temporal region to the occipito-temporal gyrus (fusiform gyrus). B2 (4). Tau propagates further anteriorly into the frontal lobe and laterally to the inferior and middle temporal cortex. Fascia dentata and CA4 region of the hippocampus may be affected in this phase. B3 (5). Tau aggregates extend into secondary cortical areas including motor, sensory, and visual association cortices. From the posterior cingulate gyrus, they spread posteriorly and affect the precuneus in the medial parietal lobe. Furthermore, tau encompasses other anatomical areas, including superior temporal cortex and pars compacta of substantia nigra. B3 (6). In the last phase, tau spreads into primary motor, sensory, and visual cortices [43].

The B-score is derived from the Braak staging system (Figure 2) [42,43]. It is also scored on a 4-point scale of 0–3 and is based on a progression of abnormal tau protein in the form of NFTs, which are an integral part of AD pathology, and dystrophic neurites found in the periphery of NPs. As with the A-score, “0” denotes an absence of tau from immunohistochemical staining. Subsequent B-scores are based on Braak staging, beginning with the transentorhinal cortex (Stage I) and expanding to the entorhinal cortex and hippocampus (Stage II). Tau deposition subsequently involves the temporal neocortex, including occipito-temporal and lingual gyrus (Stage III), and more laterally expands to involve the middle temporal gyrus (Stage IV). Finally, tau aggregates spread to the remaining cortex (Stage V–VI), and the basal ganglia can also be involved in some cases. Accordingly, stage I–II, stage III–IV, and stage V–VI are scored as B1, B2, and B3, respectively [38].

**Figure 3:**
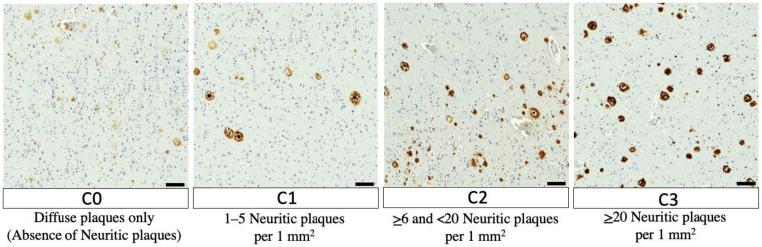
Updated CERAD scoring [38], Scale bars are 100 μm. Examples of senile plaque density for each CERAD score on immunohistochemistry using mouse anti-human β-amyloid (6F3D) antibody [44].

The final component of the ABC scoring system, the C-score (Figure 3), is based on the NP density in the neocortex according to the CERAD protocol [39]. Very simply, the density of NPs is scored as C0, C1, C2, or C3, which denote absent, sparse, moderate, or frequent NPs, respectively; templates from CERAD papers can be used as guides for this assessment [39]. It is important to note that diffuse plaques are not counted in this density assessment. This last component of the scoring system accounts for the importance of NP density as both a marker for AD neuropathology and an independent predictor of cognitive status antemortem [16].

The final step is determining the likelihood that the neuropathologic findings explain the antemortem clinical syndrome based on the assigned ABC score as “not” (i.e., not AD), “low”, “intermediate”, or “high” according to Montine et al. [38] (Table 1). For example, the highest score, A3B3C3, corresponds to a “high” likelihood that the Alzheimer’s disease neuropathologic changes explain the clinical syndrome, whereas a score of A1B2C1 corresponds to a “low” likelihood.

**Table 1:** ABC Scoring (extracted from Montine et al.) [38].

AD Pathology		B; NFT Braak Stage ^a^		
A; β-Amyloid ^b^ Thal Phase > Score	C; CERAD ^c^ Plaque Density	0 or 1	2	3
0	0	Not ^d^	Not ^d^	Not ^d^
1	0 or 1	Low	Low	Low ^e^
	2 or 3 ^f^	Low	Intermediate	Intermediate ^e^
2	Any C	Low ^g^	Intermediate	Intermediate ^e^
3	0 or 1	Low ^g^	Intermediate	Intermediate ^e^
	2 or 3	Low ^g^	Intermediate	High

ABC scoring combines different possibilities of AD neuropathologic changes and generates a qualitative score including “Not” (i.e., not AD), “Low”, “Intermediate”, and “High”. Intermediate or High better justify the AD diagnosis for clinical dementia [38]. ^a^ NFT Braak stage [42,43], ^b^ Thal beta-amyloid plaque phase/score [40], and ^c^ CERAD senile plaque score [39] are essential for ABC scoring. ^d^ Presence of NFTs in medial temporal regions without Aβ or senile plaques can be due to aging. It can also be seen in subjects with mild cognitive impairment or in those whose cognitive impairment is due to other causes rather than AD [45]. ^e^ In cases with extensive NFTs and low level of amyloid plaques, other tauopathies should be considered. ^f^ High density of senile plaques with a low Thal phase warrants reassessment of senile/diffuse plaques, considering the contribution of other diseases to cognitive impairment or dementia. ^g^ In the setting of high Aβ burden with low Braak stage, co-morbidities such as Lewy body disease, vascular brain injury, or hippocampal sclerosis can be considered. Additional sections should be studied to evaluate other non-AD pathologies [38].

## 3. Alzheimer’s Dementia Clinical Subtypes

Historically, AD has been characterized by progressive impairment in episodic memory, executive function, language, and visuospatial function, with the most prominent deficits in memory [25,46,47]. Thanks to new PET imaging techniques, it is possible to map the spread of tau in different brain regions of living subjects. In vivo observations in these imaging studies have been shown to have significant compatibility with classic Braak staging [48,49,50,51,52]. Since the advent of CSF biomarkers, molecular neuroimaging, and neuropsychological evaluations, four distinct subtypes have emerged: limbic-predominant, medial temporal lobe (MTL)-sparing, posterior, and lateral temporal [53]. These subtypes are based on in vivo tau deposition and have a strong correlation between corticolimbic network involvement and expected clinical presentation and progression. The limbic subtype most closely resembles the classical amnestic predominant presentation and progression of AD. On average, patients with the limbic subtype have worse delayed-recall memory but initially perform better across other cognitive domains compared to other subtypes. Tau progression in the limbic subtype starts in the entorhinal cortex and follows the typical pattern noted by the Braak staging scheme. This group is also more likely to include APOE4 carriers and have an older age of onset. Patients with the MTL-sparing subtype tend to be younger at onset, with an overall higher tau burden in the frontoparietal networks and more prominent deficits in executive function and working memory [53]. New reports demonstrate evidence of neuropathologic findings seen in the MTL-sparing subtype [54] showing a possible different pathway of pTAU pathology that first involves parietal cortex, followed by limbic structures. The posterior subtype shows prominent deficits in visuospatial functions and corresponds with the clinical phenotype of posterior cortical atrophy [47]. Finally, the lateral temporal subtype, particularly when lateralized to the dominant hemisphere, is characterized by more prominent language involvement and corresponds with the clinical phenotype of logopenic variant primary progressive aphasia. Taken together, these subtypes explain much of the heterogeneity seen among AD patients and have significant implications for patient counseling on prognosis, symptom progression, and targeted therapy in the future.

## 4. Discussion

Alzheimer’s disease continues to be the sixth-leading cause of death in the United States, with total payments to health care, long-term care, and hospice care estimated to be $355 billion (AD facts and figures 2021) [55]. Therapeutic research efforts have focused on amyloid-β protein and tau aggregates, emphasizing the importance of an accurate postmortem neuropathologic diagnosis of Alzheimer’s disease to support antemortem biomarkers and clinical diagnoses [2,56]. The ability to establish a confident antemortem diagnosis has significant implications for future research and allows families to make informed decisions for the future. Furthermore, the addition of Alzheimer’s dementia clinical phenotypes has put a spotlight on specific patterns of pathological change. It is hoped that this summary of the ABC scoring system will be a useful reference for AD scientists, clinicians, neurologists, neuropathologists, and general pathologists and provide a better understanding of the diagnosis and staging of Alzheimer’s disease with efficiency and accuracy. Additionally, this work is aimed at assisting scientists working on preclinical models of AD to ensure that their work can be translated to the clinic.

## Data Availability

No new data were created or analyzed in this study. Data sharing is not applicable to this article.
